# Sustainable Pharmacy: Piloting a Session on Pharmaceuticals, Climate Change, and Sustainability within a U.S. Pharmacy Curriculum

**DOI:** 10.24926/iip.v8i4.929

**Published:** 2017-10-12

**Authors:** Katherine Gruenberg, Dorie Apollonio, Conan MacDougall, Tina Brock

**Affiliations:** a University of California San Francisco School of Pharmacy; b Monash University Faculty of Pharmacy and Pharmaceutical Sciences

**Keywords:** Environmental impact, Sustainability, Pharmacy, Education, Pharmaceuticals

## Abstract

**Objective:**

To design and assess an innovative session for pharmacy students that addresses the role of pharmaceuticals with climate change and sustainability.

**Innovation:**

One hundred and sixteen third-year students at the University of California, San Francisco School of Pharmacy participated during their required Health Policy course. This 3-hour session included guided pre-course activities, an interactive lecture, a panel of healthcare professionals discussing complex decision-making and small group case-based learning. Curricular assessment was conducted through pre-/post-test measures of knowledge acquisition, student evaluations, and course projects.

**Critical Analysis:**

One hundred and two students (response rate 88%) completed the pre-test and 115 students (response rate 99%) completed the post-test assessment. We identified a significant increase in the proportion of correct answers on post-test questions addressing drug disposal legislation (75% pre-test vs 91% post-test, p=0.002) and the predicted effects of climate change on health (55% pre-test vs 90% post-test, p < 0.001). The session was also well received; average student evaluation scores were above 4 in all areas of course evaluation (where 5=ideal). In addition, 17% of student groups (relative to 0% in 2015) proposed a sustainability-related policy as their final coursework project.

**Next Steps:**

The development and implementation of this brief session resulted in knowledge gain and favorable student response. This project is feasible for other Schools of Pharmacy to adapt and implement.

## Introduction

Climate change is described as ‘the greatest global health opportunity of the 21^st^ century’. As a result of climate change, a greater incidence of vector-borne diseases, heat stress, and malnutrition is anticipated.^1^ Additionally, certain co-morbid populations will likely be disproportionally affected by extreme environmental conditions.^2^ For example, a patient with asthma may experience more frequent disease exacerbations due to poor air quality.

Increased disease burden will be met with greater pharmaceutical utilization and demand. Pharmaceuticals impact the environment from drug development to disposal. In 2007, the U.S. healthcare system contributed an estimated 7% of total carbon dioxide emissions, of which 39% was attributed to hospitals and 14% to pharmaceutical companies.^3^ In addition to the carbon footprint of pharmaceutical companies, inappropriate pharmaceutical disposal leads to contamination of water and bioaccumulation in wildlife. Historically, waste treatment was expected to degrade any active pharmaceutical compounds to an acceptable risk level. More recently, however, reports of feminized fish have been attributed to water contaminated with estrogen-containing contraceptives.^4^ Increased pharmaceutical consumption will only amplify the environmental consequences of the pharmaceutical lifecycle. It is imperative for healthcare trainees and professionals worldwide to be aware of these issues and to advocate for ‘greener’ pharmaceutical production and disposal. Some countries have responded to this need by introducing formal training for students studying medicine and nursing.^5,6^ Recognizing the scope of this issue within the U.S., the CDC has emphasized training of “…public health staff to respond to the health threats posed by climate change” as a priority health action.^7^ Like physicians and nurses, pharmacists have a vital role to play in climate-related prevention, education, and policy. Within pharmacy education, the 2013 Center for the Advancement of Pharmacy Education (CAPE) Educational Outcomes support teaching environmental health topics as part of, “evaluating personal, social, economic, and environmental conditions to maximize health and wellness”.^8^ However, pharmacy curricula that address these concepts have not been identified within the literature.

## Statement of Innovation

Recognizing the crucial need to include these topics within pharmacy education, we designed, implemented, and evaluated a three-hour session at The University of California, San Francisco (UCSF) School of Pharmacy. Our educational materials focused on the relationships between climate change, sustainability, and pharmaceuticals. We predicted this session would increase student knowledge in these areas.

## The Innovation

The UCSF School of Pharmacy offers a required health policy course for third-year pharmacy students. Each session meets for three hours per week for a total of 10 weeks between September and December each year. The decision to incorporate this session within the existing health policy course was based on a 2015 drug disposal policy in San Francisco that mandated citywide pharmaceutical “take back” and the creation of a campus-wide sustainability initiative in 2008. Our goals were to: (1) introduce and expand student knowledge of the relationships between climate change, sustainability, and healthcare and (2) inspire pharmacist involvement through health policy awareness and action. We emphasized a stakeholder-approach when analyzing current practices and identifying opportunities for sustainable change.

One week prior to this session, students were invited to take a multiple-choice pre-test assessment of knowledge via an anonymous Qualtrics™ (Qualtrics, Provo, UT) online link. These questions addressed four key concepts related to the session goals: (1) the impact of climate change on health, (2) options for controlled substance disposal, (3) local drug disposal policy, and (4) available environmental health web resources.

Just prior to the session, students were asked to work in small groups to complete pre-course readings and corresponding questions. The goal of this work was to introduce the direct effects of pharmaceuticals on the environment (Appendix 1). Each group responded to the questions in an online forum monitored by the session instructor. Selected student responses were later incorporated within the large group didactic session.

**Figure 1:**
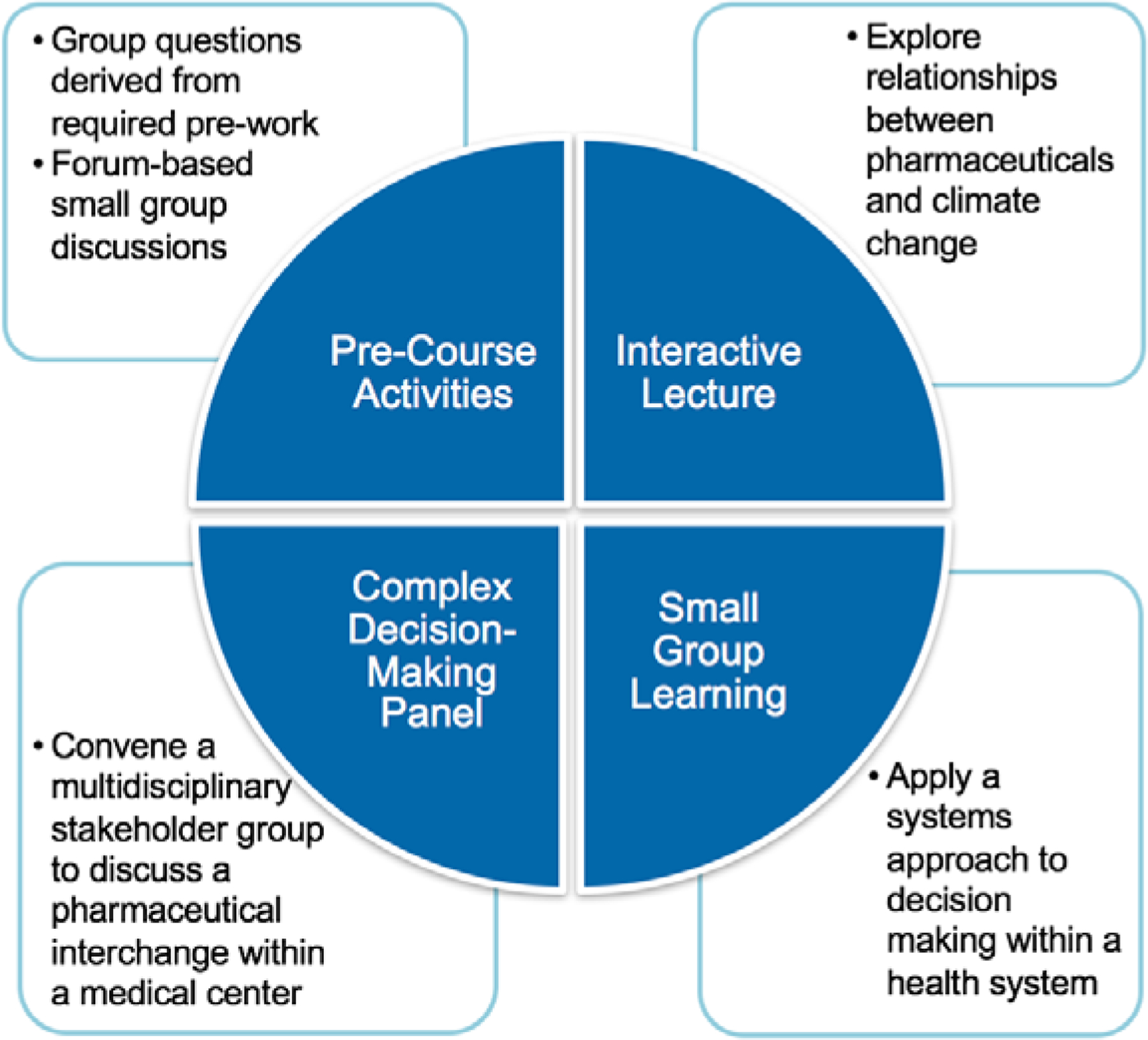
Summary of Session Components

**Figure 2:**
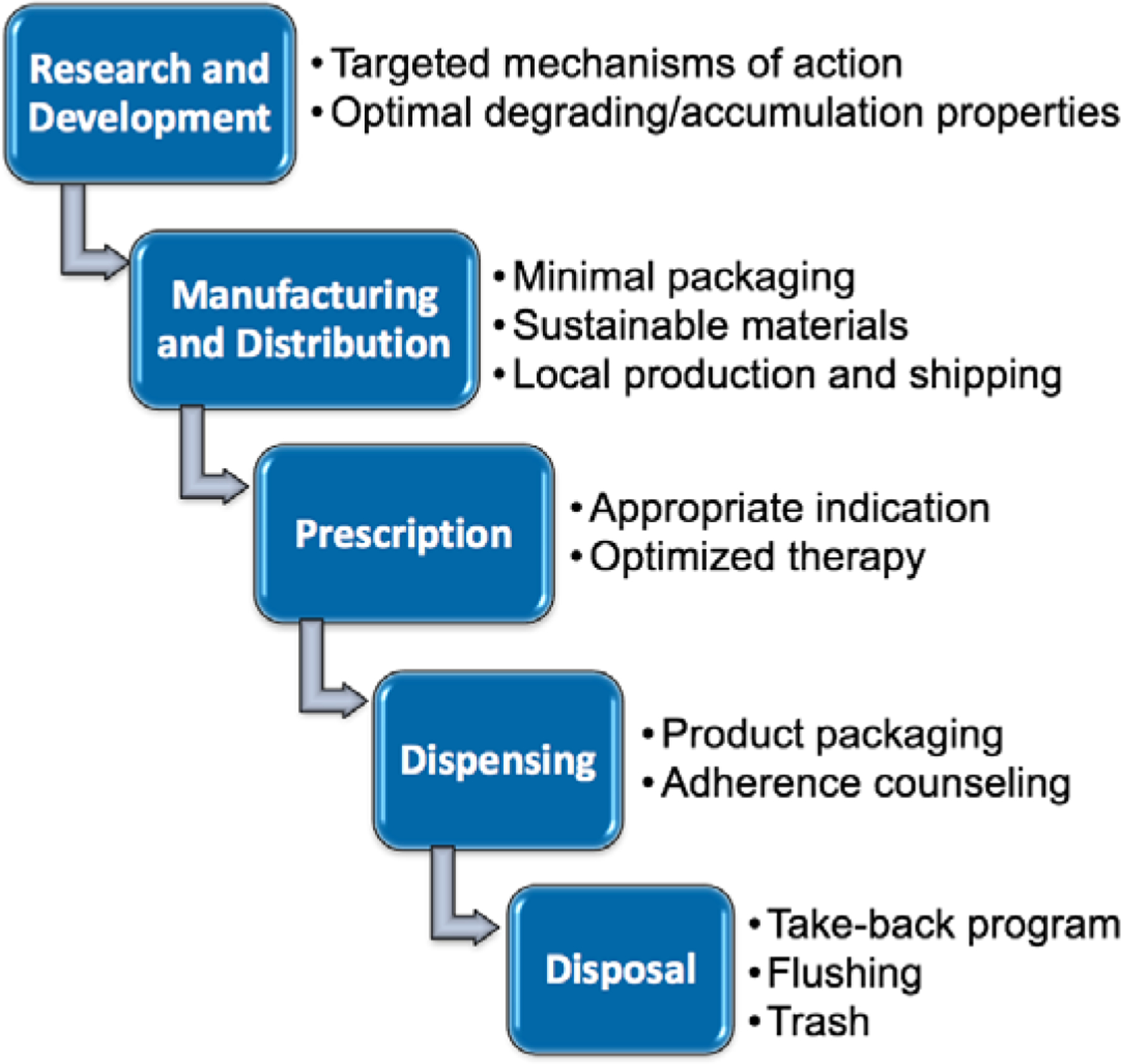
Sustainable Pharmaceutical Life Cycle

We employed a variety of active teaching methods during the class (Figure 1). Students first engaged in a large group discussion that reinforced and expanded upon concepts addressed in the pre-coursework. We encouraged participation through an anonymous audience response system, Poll Everywhere^®^ (PollEverywhere, San Francisco, CA). Later in the session, students worked in small groups to examine the environmental impact of pharmaceuticals from drug development to disposal (Figure 2). To provide clinical context to our discussion, a patient case was introduced. Students were instructed to evaluate the environmental impact of amoxicillin prescribed for a pediatric case of possible otitis media. During this exercise, students applied their knowledge of pharmaceutical chemistry, health system supply chain management, antimicrobial stewardship, dispensing preferences, and medication disposal options.

Following this conversation, another patient case involving a nebulizer to inhaler pharmaceutical substitution program was presented. This case was adapted from an actual policy adopted at UCSF Medical Center in 2013. The policy required all eligible inpatients receiving nebulized bronchodilators to be transitioned to inhalers within 24 hours of initial nebulized administration. This policy was chosen to highlight the unintended environmental impact of increased inhaler waste.

After the students reflected upon the case, a panel of professionals in medicine, pharmacy, respiratory therapy, and environmental sustainability were invited to discuss the nebulizer-to-inhaler policy (Appendix 1). The panelists modeled a collaborative approach to balancing resource utilization, operations, and costs with quality care. The patient perspective, represented by an actor, generated a dialogue around the role of patient beliefs within health system policy development. The panel was videotaped for use in future sessions and for external dissemination (https://vimeo.com/230639355). At the conclusion of the panel, students re-grouped to consider whether they would have adopted this policy given the environmental implications alongside each stakeholder perspective.

Students were then invited to take the same 4-question multiple-choice assessment via an anonymous Qualtrics™ (Qualtrics, Provo, UT) online link. Assessment was conducted through pre-/post-test measures of knowledge acquisition, student evaluations, and course projects. Test responses were exported and analyzed within Microsoft® Excel v. 15.35 for Mac (Redmond, Washington). Statistical significance was defined *a priori* when p < 0.05. Since participant responses were anonymous and unpaired, intergroup pre-/post-test responses were analyzed using Chi-squared test. This pilot study was approved through exemption by the UCSF Institutional Review Board (IRB#16–20893).

## Critical Analysis

One hundred and sixteen third-year pharmacy students attended this course in 2016. One hundred and two students (response rate 88%) completed the pre-test and 115 students (response rate 99%) completed the post-test. We identified a significant increase in the proportion of correct answers on post-test questions addressing drug disposal legislation (75% pre-test vs 91% post-test, p= 0.002) and the predicted effects of climate change on health (55% pre-test vs 90% post-test, p<0.001). Questions addressing options for controlled drug disposal (68% pre-test vs 74% post-test, p=0.31) and informational resources (43% pre-test vs 50% post-test, p=0.28) showed a trend towards increased knowledge, but these results were not statistically significant. We suggest a few reasons for these results. First, there are competing interests between environmental priorities and diversion of controlled substances. The DEA and FDA support drug take back programs, but also the disposal of controlled substances in the trash or toilet to minimize diversion. Therefore, student responses varied between these disposal methods. Regarding the informational resource question, a list of references was provided at the close of the session, but it was not emphasized within the learning objectives. In the future, it would be beneficial to invite a deeper conversation about controlled substance disposal and to spend more time emphasizing resources students and patients can consult for additional information.

The final course assignment required student groups to present a health-related policy inspired by one of the course sessions. Five of 30 student groups (17%), relative to 0% in 2015, proposed a sustainability-related policy for this assignment. The primary themes of these policies included: drug disposal information on pharmaceutical package inserts, reduction of inhaler hazardous waste, and drug take-back programs.

At the conclusion of the term, 31% of students were randomly assigned to complete teaching evaluations by the UCSF Office of Student and Curricular Affairs. The results of these evaluations suggest the session was well-received. Average student evaluation scores were above 4 (Likert Scale, where 1=strongly disagree and 5=strongly agree) in all areas of course evaluation. The evaluation themes and corresponding student evaluation scores (mean ± SD) were as follows: learning session was well-organized (4.17 ± 0.74) and participation was encouraged (4.11 ± 0.75). Student comments also positively reflected the overall value of the course. One student remarked, “I really appreciate that this topic gets brought up because we often don’t think about what happens to unused medications.” Another student commented, “They [session materials] were easy to follow and gave students insight about the importance of sustainability with pharmacy practice”.

## Discussion

The environmental impact of pharmaceuticals is well established, yet curricula addressing this topic have not been identified in the literature. Before the implementation of this session, only half of UCSF pharmacy students were aware of the effects of climate change on health. However, a recent survey of 1,275 American adults revealed that 64% of respondents thought global warming has negative health implications and, amongst various sources, primary care doctors were designated as the most trusted source of information, before the Environmental Protection Agency, World Health Organization, and Centers for Disease Control and Prevention.^9^ This knowledge disconnect and public trust in healthcare professionals emphasizes the need to incorporate these topics within health professions education. The International Pharmaceutical Federation has taken a professional stance on this topic by issuing a policy statement for implementation of environmentally sustainable pharmacy practices and education.^10^ While this is a progressive policy, implementation is lagging within the U.S.

Pharmacists have significant potential to mitigate the environmental risks of pharmaceuticals. In Sweden, a drug database (www.fass.se) has been developed to classify the environmental risk of medications.^11^ The data are provided voluntarily by pharmaceutical companies and then reviewed by the Swedish Environmental Research Institute, an independent organization. Each drug is evaluated on environmental hazard, persistence, and bioaccumulation risk. For example, data from AstraZeneca reveal that amoxicillin causes moderate environmental risk, is potentially persistent, and has low potential for bioaccumulation.^12^ This information is publicly available and could be incorporated into the therapeutic decision-making process. In fact, we used this database during our otitis media case to discuss the value of such a tool in the prescribing process. All other things being equal, selecting a drug with reduced environmental impact is preferred. Within the United States, the National Environmental Policy Act of 1969 requires the FDA to evaluate the environmental impact of drugs and biologics. Pharmaceutical companies must submit an environmental assessment or claim an exclusion when submitting applications for new drugs, abbreviated new drugs, biologic licenses, or investigational new drugs.^13^ With this information, the possibility of creating a similar database within the United States is feasible.

However, until such a tool is available and accepted within the U.S., pharmacists can emphasize proper pharmaceutical disposal through education of healthcare providers and patients. San Francisco created a Safe Drug Disposal Stewardship Ordinance in 2015, which requires pharmaceutical companies to facilitate safe drug disposal throughout the city.^14^ Volunteer collectors include law enforcement, local pharmacies, and mail-back programs. As of June 2017, the California Board of Pharmacy has finalized the regulation of pharmacies and healthcare settings with onsite pharmacies that provide pharmaceutical take-back services.^15^ Promoting awareness of these services to pharmacists, prescribers, and patients is an important step towards reducing the environmental impact of pharmaceuticals.

## Next Steps

Pharmacy curricula addressing the interaction between climate change and pharmaceuticals are pertinent, yet lacking within the United States.^2^ The development and implementation of this brief session resulted in knowledge enhancement and favorable student response.

We presented our session internally to the UCSF Carbon Neutrality Initiative Faculty group in December 2016 (http://sustainability.ucsf.edu/3.656) and in modified format to the pharmacists at the UCSF Medical Center in December 2016. We also shared this internationally at the Monash University Pharmacy Education Symposium in June 2017 (https://www.monash.edu/pharm/about/events/education-symposium), where we identified potential international collaborators.

This session will be used again in the 2017 Health Policy course at UCSF. Looking forward, we hope to collaborate with other U.S. pharmacy programs to strengthen and extend this work. To facilitate the adaptation of this session by other schools, our teaching materials are available for use through Creative Commons licensing at pharmacademy.org. An abbreviated version of these materials is available to preview in Appendix 1.

## Appendix 1: Preview of course materials

### Full materials are on pharmacademy.org.

#### Pre-Coursework:

##### Review the following links/articles and answer group questions:

- “Drugs in the Water”: https://www.health.harvard.edu/newsletter_article/drugs-in-the-water
- Lubick N. Drugs in the environment: do pharmaceutical take-back programs make a difference? Environ Health Perspect.2010;118(5):A210–4.
- Explore the hospital on Tox Town: https://www.toxtown.nlm.nih.gov/scenes/city/scene.php

##### Selected Group Discussion Questions

1. Based on your reading of ‘Drugs in the Environment’ and ‘Drugs in the Water’, describe the pros and cons of the three pharmaceutical disposal methods (take-back programs, flushing, and trash disposal). Then describe how you would counsel a patient to dispose of their pharmaceuticals.
2. Consider the ways in which hospital or community pharmacies create unnecessary waste and develop an actionable plan to reduce the pharmacy’s environmental impact.
3. Based on your readings, brainstorm potential legislation that could be implemented to address the environmental impact of pharmaceuticals.

#### In class: Complex Decision-making Panel (https://vimeo.com/230639355)

**Table:**

Stakeholder	Panelist	Perspective
Administration	Catherine Lau, MD	Value
Environment	Gail Lee, REHS, MSEM, HEM	Sustainability
Clinician - pharmacy	Fanny Li, PharmD, BCPS, BCCCP	Formulary
Clinician - respiratory therapy	Brian Daniel, RCP, RRT	Workflow
Patient	Evans Whitaker, MD, MLIS	Convenience

#### Panelist Remarks:

1. From your professional perspective, what would be the benefit of implementing the ‘Nebs No More After 24’ proposal?
2. What are some considerations or concerns you have regarding this proposal specifically related to your role?
3. Do changes in the political climate (within the health-system or within the wider world) influence this decision? If so, how?

## References

[1] Watts N, Adger W, Agnolucci P, Health and climate change: Policy responses to protect public health. Lancet. 2015;386(10006):1861–914. doi:10.1016/S0140-6736(15)60854-6 26111439

[2] Maxwell J, Blashki G. Teaching about climate change in medical education: An opportunity. J Public Health Res. 2016;5(1):673. doi:10.4081/jphr.2016.673 27190980 PMC4856872

[3] Chung J, Meltzer D. Estimate of the carbon footprint of the US health care sector. JAMA. 2009;302(18):1970–2. doi:10.1001/jama.2009.1610 19903917

[4] Thomas F, Depledge M. Medicine ‘misuse’: Implications for health and environmental sustainability. Soc Sci Med. 2015;143:81–7. doi:10.1016/j.socscimed.2015.08.028 26344126

[5] Bajgoric S, Appiah J, Wass V, Shelton C. Sustainability in clinical skills teaching. Clin Teach. 2014;11(4):243–6. doi:10.1111/tct.12141 24917089

[6] Richardson J, Grose J, Doman M, Kelsey J. The use of evidence-informed sustainability scenarios in the nursing curriculum: Development and evaluation of teaching methods. Nurse Educ Today. 2014;34(4):490–3. doi:10.1016/j.nedt.2013.07.007 23948087

[7] Centers for Disease Control and Prevention. Climate and health CDC Policy. https://www.cdc.gov/climateandhealth/policy.htm Published December 14, 2009. Updated December 22, 2014. Accessed August 17, 2017.

[8] Medina M, Plaza C, Stowe C, Center for the Advancement of Pharmacy Education 2013 Educational Outcomes. Am J Pharm Educ. 2013;77(8):Article 162. doi:10.5688/ajpe778162. 24159203 PMC3806946

[9] Maibach EW, Kreslake JM, Roser-renouf C, Rosenthal S, Feinberg G, Leiserowitz AA. Do Americans understand that global warming is harmful to human health? Evidence from a national survey. Ann Glob Health. 2015;81(3):396–409. doi:10.1016/j.aogh.2015.08.010 26615074

[10] International Pharmaceutical Federation. FIP Statement of Policy- Environmentally Sustainable Pharmacy Practice: Green Pharmacy. The Hague, The Neatherlands: FIP, 2016.

[11] Wennmalm A, Gunnarsson B. Pharmaceutical management through environmental product labeling in Sweden. Environ Int. 2009;35(5):775–7. doi:10.1016/j.envint.2008.12.008 19193440

[12] The Swedish Association of the Pharmaceutical Industry. Amoxicillin Environmental Impact Information. Fass Website. http://www.fass.se/LIF/product?userType=0&nplId=20050717000026&docType=78&scrollTopPosition=387&docTypeDynTab=78. Accessed August 21, 2017.

[13] US Department of Health and Human Services. Guidance for Industry Environmental Assessment of Human Drug and Biologics Applications. https://www.fda.gov/downloads/Drugs/Guidances/ucm070561.pdf. Revised July 1998. Accessed August 21, 2017.

[14] San Francisco Department of the Environment Regulation #SFE-10–01-SDDSO. Regulations Implementing the Safe Drug Disposal Stewardship Ordinance (31–15) https://sfenvironment.org/sites/default/files/editor-uploads/toxics/pdf/sfe_th_regulations_safe_drug_disposal_stewardship_ordinance_july_25.pdf. Effective July 25, 2016. Accessed August 21, 2017.

[15] California Board of Pharmacy. Order of Adoption Prescription Drug Take-Back. http://www.pharmacy.ca.gov/laws_regs/1776_oa.pdf. Effective June 8, 2017. Accessed August 21, 2017.

